# The Virome of Healthy Honey Bee Colonies: Ubiquitous Occurrence of Known and New Viruses in Bee Populations

**DOI:** 10.1128/msystems.00072-22

**Published:** 2022-05-09

**Authors:** Dominika Kadlečková, Ruth Tachezy, Tomáš Erban, Ward Deboutte, Jaroslav Nunvář, Martina Saláková, Jelle Matthijnssens

**Affiliations:** a Department of Genetics and Microbiology, Faculty of Science BIOCEV, Charles University, Prumyslova, Vestec, Czech Republic; b Crop Research Institute, Prague, Czech Republic; c Max Planck Institute for Immunobiology and Epigenetics, Freiburg, Germany; d KU Leuven—Rega Institute for Medical Research, Laboratory of Viral Metagenomics, Leuven, Belgium; Vanderbilt University

**Keywords:** *Apis mellifera*, *Picornavirales*, rhabdovirus, Lake Sinai virus, metagenomics, viruses

## Abstract

Honey bees are globally important pollinators threatened by many different pathogens, including viruses. We investigated the virome of honey bees collected at the end of the beekeeping season (August/September) in Czechia, a Central European country. Samples were examined in biological replicates to assess the homogeneity, stability, and composition of the virome inside a single hive. By choice of healthy workers from colonies, where Varroa destructor was under control, we could identify ubiquitous bee viruses. Deformed wing virus (DWV) was highly prevalent, even though the bees were healthy, without any noticeable disease signs. The overall virome composition (consisting of honey bee-, plant-, and bacterium-infecting viruses) was driven primarily by the hive and its location. However, honey bee-specific viruses showed an uneven distribution within the same hive. In addition, our results point to an unusual cooccurrence between two rhabdoviruses and reveal the presence of five distinct lineages of Lake Sinai viruses (LSVs) clustering with other LSV strains described globally. Comparison of our results with the virome of Australian honey bees, the last truly *Varroa-* and DWV-free population, showed a strong difference with respect to DWV and a set of diverse members of the *Picornavirales*, of which the latter were absent in our samples. We hypothesize that the occurrence of DWV introduced by *Varroa* strongly affects the virome structure despite the mite being under control.

**IMPORTANCE** The Western honey bee, Apis mellifera, is a vital part of our ecosystem as well as cultural heritage. Annual colony losses endanger beekeeping. In this study, we examined healthy bees from the heart of Central Europe, where honey bee colonies have been commonly affected by varroosis over 5 decades. Our virome analysis showed the presence of ubiquitous viruses in colonies where the mite *Varroa destructor* was under control and no honey bee disease signs were observed. Compared to previous studies, an important part of our study was the analysis of multiple replicates from individual hives. Our overall results indicate that the virome structure (including bee-infecting viruses, plant-infecting viruses, and bacteriophages) is stable within hives; however, the bee-infecting viruses varied largely within interhive replicates, suggesting variation of honey bee viruses within individual bees. Of interest was the striking difference between the viromes of our 39 pools and 9 pools of honey bee viromes previously analyzed in Australia. It could be suggested that *Varroa* not only affects DWV spread in bee colonies but also affects diverse members of the *Picornavirales*, which were strongly decreased in Czech bees compared to the *Varroa*- and DWV-naive Australian bees.

## INTRODUCTION

The European honey bee, Apis mellifera Linnaeus 1758, is used for the production of honey, propolis, beeswax, venom, pollen, and royal jelly (1). However, the most crucial beneficial feature of honey bees lies in pollination in both agricultural (2, 3) and natural (4) habitats. Annual colony losses jeopardize these benefits provided by honey bees (5). In temperate zones of Europe, the main colony losses occur over winter and are thus referred to as wintering losses (6). Another problem, not yet fully understood, is colony losses in the United States, also known as colony collapse disorder (CCD) (7). This phenomenon is probably due to a combination of several factors, mainly Varroa destructor (8), other viral pathogens, and their interaction (7, 9). Conservation of honey bees is difficult, especially in countries where the density of managed colonies is very high, and this is precisely the case in Czechia, in the heart of Central Europe (10).

The global spread of V. destructor has had a severe effect on the transmission and virulence of certain honey bee viruses such as deformed wing virus (DWV); DWV variant B (DWV-B), also described as Varroa destructor virus 1 (11, 12); and viruses belonging to the acute-Kashmir-Israeli complex (13–15). However, without high mite infestations, DWV infections are often benign or asymptomatic. Important from this point of view is the presence of diverse viruses in Australia, where honey bees are free of both *V. destructor* and DWV, although some viruses from the *Picornavirales* order can be found (16). Interestingly, the interaction between viruses and the mite can affect virus strain distribution, as described previously for DWV (17–20). The DWV-A/B strain ratio is affected by the level of mite infestation in a colony (21). Another parasite, Nosema ceranae, has been shown to aggravate black queen cell virus (BQCV) infection (22). However, the synergistic effect of N. ceranae in combination with different viruses such as DWV was negated (23). Thus, for other known or newly identified viruses, similar or unexpected interactions may exist. Various nonviral pathogens may play an important role in the prevalence and severity of diseases.

Until lately, honey bee virus research focused mainly on 23 described viral species, as reviewed in 2015 (24). In the last few years, more viruses that demonstrably or presumably infect honey bees were discovered due to the increased use of next-generation sequencing (NGS) technologies (16, 25, 26). Moreover, it was recently shown that the honey bee gut virome contains many bacteriophages (27, 28). Previous knowledge was limited to phages from pathogens such as Paenibacillus larvae (27). In contrast to the best-characterized bee-infecting viruses, which belong to the *Picornavirales* or other positive-sense single-stranded RNA (+ssRNA) virus groups (e.g., *Iflaviridae* and *Dicistroviridae*), some novel viruses belonging to viral families like the *Rhabdoviridae* or *Orthomyxoviridae* have recently been reported in honey bees (29, 30) and the parasite *V. destructor* (30). Most infections with these novel viruses are not yet known to manifest symptomatically but could impact colony health through fitness costs, even though subtle, for the host and/or through interactions with the host and other pathogens/parasites associated with honey bees. Furthermore, the spread of viral infections from honey bees to wild pollinators is also of great concern (31, 32).

In this study, we explored the diversity and composition of the virome in honey bees from healthy colonies from beekeepers breeding various honey bee genetic lines in Czechia. To see the robustness of the virome analyses, we analyzed three biological repeats from each hive. To our knowledge, this is the first such analysis performed on honey bees. We focused on the virome composition (common versus new viruses, plant viruses, and bacteriophages). In addition, we compared our results with those for nine Australian viromes from bees with no exposure to *V. destructor* or DWV.

## RESULTS

### Composition and similarity of virome samples.

NGS of 39 samples (3 replicates of 9 pooled bees from 13 colonies) yielded a total of 398,231,288 reads, with an average of 10 million reads (range, 1,920,148 to 30,170,502; median, 11,650,042) per sample containing 9 bees. The reads were classified as follows: (i) 46.66% eukaryotic, (ii) 30.98% viral, (iii) 13.14% bacterial, and (iv) 9.21% not identified as homologous to any reference sequence.

**FIG 1:**
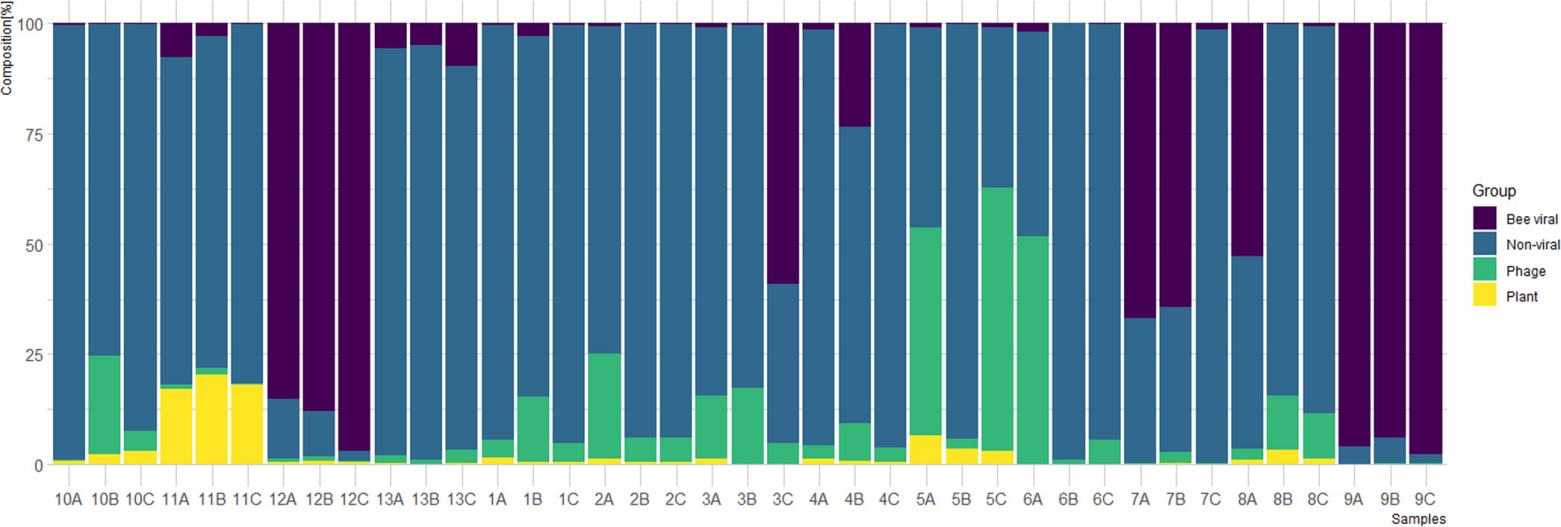
Composition of sequencing reads in all 39 analyzed samples. Each hive was analyzed in 3 independent replicates consisting of nine individual bees. The percentages of reads of different taxonomic affiliations (plant viruses, bacteriophages, bee viruses, and nonviral) are denoted in different colors.

First, we analyzed the composition of sequencing reads in each of the 39 samples by determining the proportion of reads originating from nonviral sequences (e.g., honey bee genome and bacterial microbiome), known bee viruses (33), bacteriophages, and plant viruses (Fig. 1). Although some replicates seemed to be rather consistent, notable heterogeneity was detected among several other samples (e.g., samples 3, 5, 6, 7, and 8). Next, we analyzed the taxonomic composition of the bee virome with respect to the (i) abundance and (ii) diversity of viral families (see Fig. S1 in the supplemental material).

10.1128/msystems.00072-22.1 FIG S1 Additional figures and data (map, plant heatmap, viral family distribution in the virome, comparison of samples from Australia and the rest of the world, BRV phylogeny, and additional information on k-means clustering). Download FIG S1, PDF file, 0.7 MB. Copyright © 2022 Kadlečková et al. 2022 Kadlečková et al. https://creativecommons.org/licenses/by/4.0/ This content is distributed under the terms of the Creative Commons Attribution 4.0 International license.

**FIG 2:**
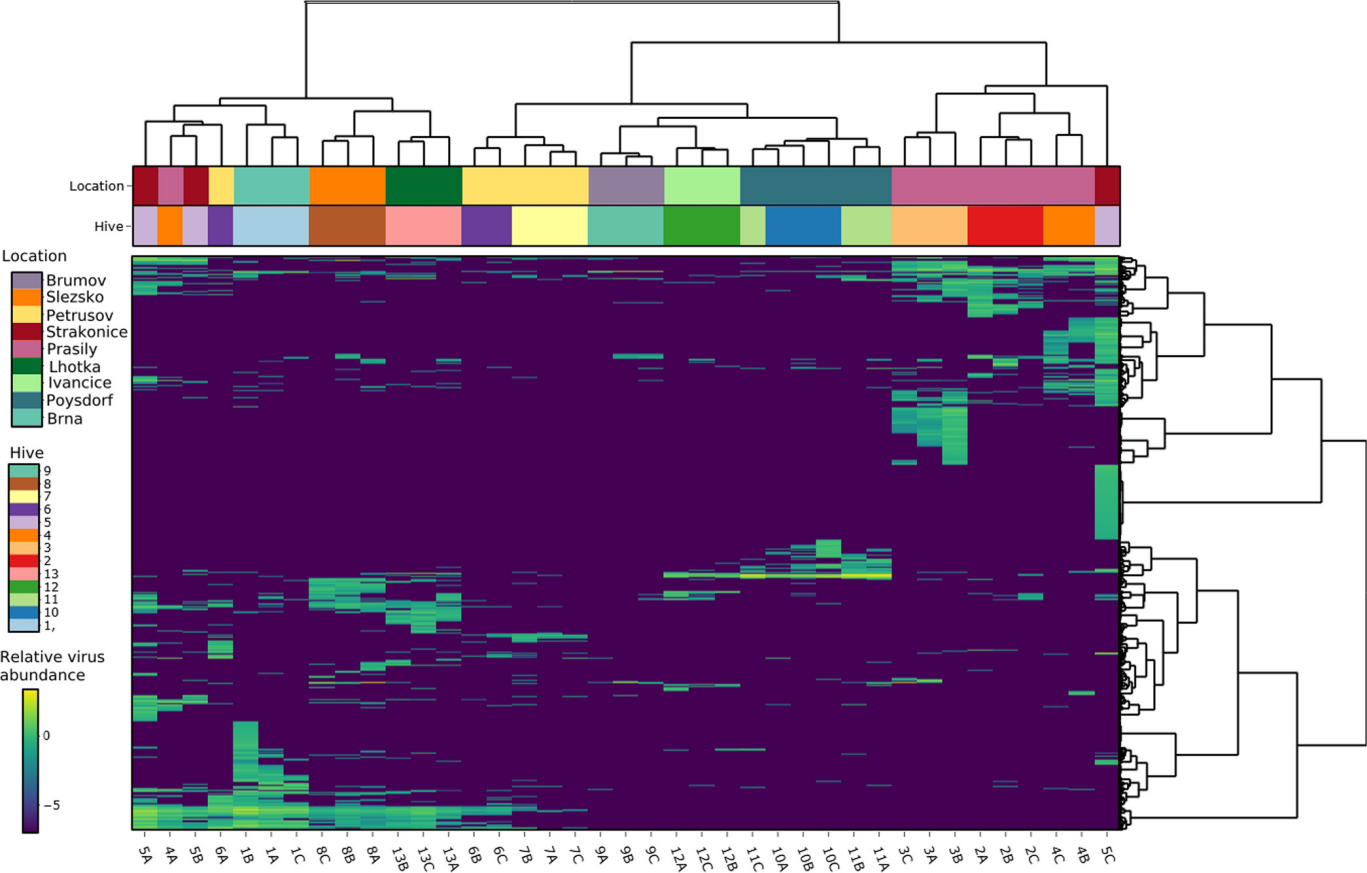
Heatmap constructed from all classified viral sequences in all 39 samples. The viral sequences are taxonomically assigned to the family or species level. Relative abundances (viruses per 1 million sequencing reads) are shown on a log_10_ scale. Samples (columns) and contigs (rows) are clustered by Ward’s minimum variance method; both columns and rows are seriated by optimal leaf ordering.

To visualize the virome similarity of the 39 samples, a heatmap was constructed from the relative abundances of all viral sequences (Fig. 2; Table S1). For almost all samples, replicates originating from the same hive clustered together and thus exhibited similar total viromes. Samples obtained from different sites (hives and apiaries) at the same location also exhibited related viromes. The Adonis test confirmed the highly significant association of virome composition with hive and location (*P* < 0.0001 by an Adonis-Bray test; *R*^2^, 0.62964 and 0.45389). k-means clustering had for k 13 (representing hives) an adjusted mutual information score (taking on values from 1 for identical to around 0 for random) of 0.14, suggesting the existence of a pattern in clustering (Fig. S1).

10.1128/msystems.00072-22.4 TABLE S1 Several sheets of raw and transformed data used to create heatmaps, also including metadata (replicate, subtype of honey bee, and location, etc.) for each sample. Download Table S1, XLSX file, 1.6 MB. Copyright © 2022 Kadlečková et al. 2022 Kadlečková et al. https://creativecommons.org/licenses/by/4.0/ This content is distributed under the terms of the Creative Commons Attribution 4.0 International license.

### Bee viruses.

**FIG 3:**
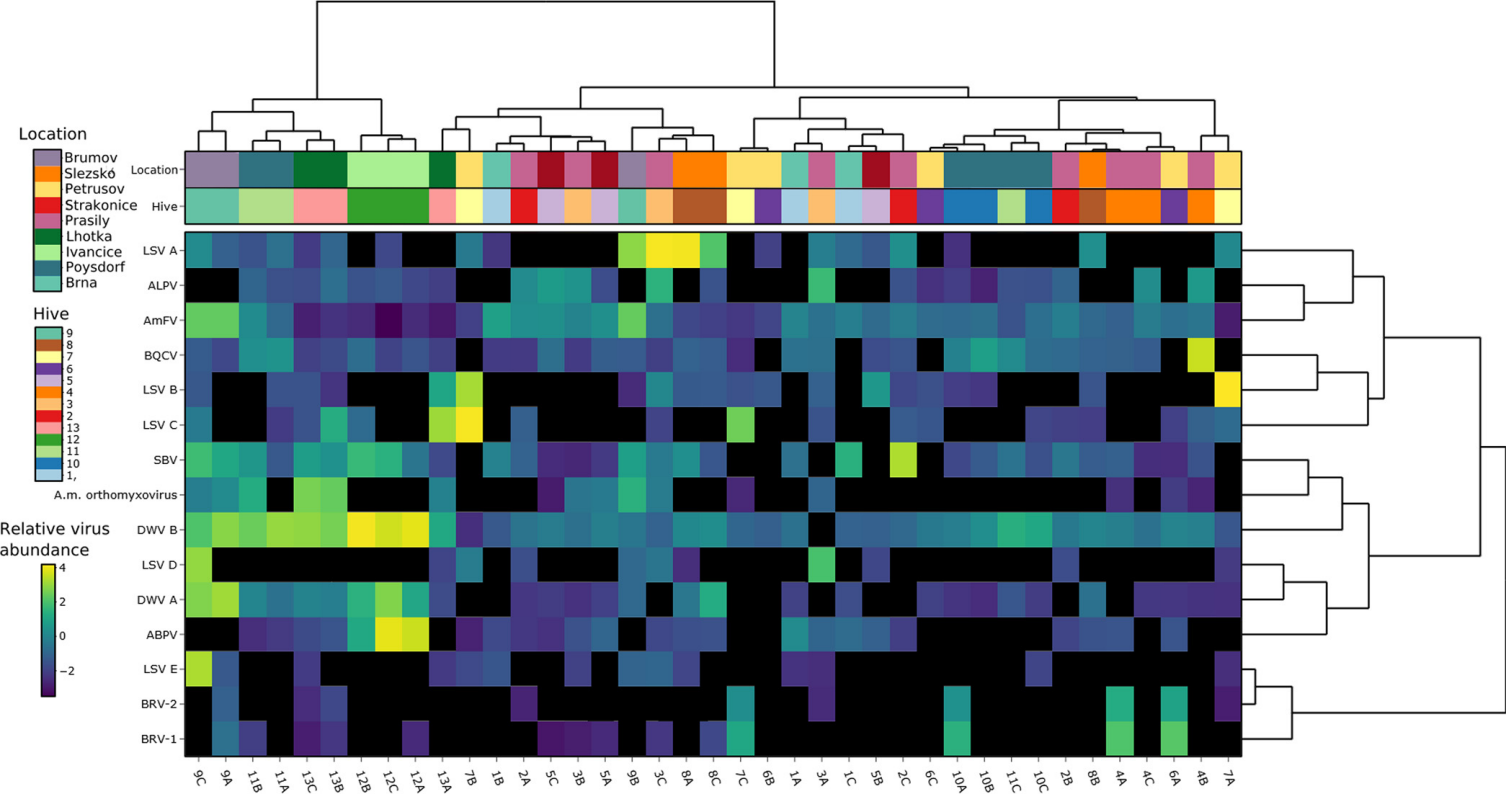
Diversity of viruses infecting honey bees in healthy bee colonies from Czechia. Relative abundances (viruses per 1 million sequencing reads, calculated from reference genome coverage [see Materials and Methods]) are shown on a log_10_ scale. Samples (columns) and viruses (rows) are clustered by Ward’s minimum variance method algorithm and seriated by optimal leaf ordering. ABPV, acute bee paralysis virus; ALPV, aphid lethal paralysis virus; AmFV, Apis mellifera filamentous virus; BRV, bee rhabdovirus; BQCV, black queen cell virus; DWV, deformed wing virus; SBV, Sacbrood virus; LSV, Lake Sinai virus.

Furthermore, we focused our analysis on eukaryotic viruses that were demonstrated or predicted to infect honey bees (33). Altogether, the analyzed samples revealed the presence of one DNA and nine RNA viruses. Besides the well-known viruses belonging to the *Dicistroviridae* and *Iflaviridae*, we found viruses belonging to the families *Rhabdoviridae* and *Orthomyxoviridae* as well as several variants of Lake Sinai virus (LSV) and the DNA virus Apis mellifera filamentous virus (AmFV) (Fig. 3).

### Overall distribution of bee viruses.

A heatmap was constructed based on the relative abundances of all detected bee-infecting viruses (Fig. 3). The most commonly present viruses were Deformed wing virus variant A (DWV-A) and variant B (DWV-B), Black queen cell virus (BQCV), Aphid lethal paralysis virus (ALPV), AmFV, and Sacbrood virus (SBV). In sharp contrast to the heatmap constructed from all viral sequences (Fig. 2), clustering between bee viruses of most replicate samples was no longer discernible (Fig. 3). We presume that the lack of geographic clustering can be attributed to the absence of bacteriophages and plant viruses (see below) in this analysis. For bee-infecting viruses, the adjusted mutual information score for k 13 (hive) was low (−0.006), further confirming that clustering between samples is absent.

High differences in abundance among the replicate samples were observed for all known bee-infecting viruses, even though each sample consisted of nine pooled bees. This implies that the pooling of nine bees per hive is not sufficient to compensate for the variability in the occurrence of viruses in individual bees. Importantly, in some samples, a single virus accounted for over 50% of the total sequencing reads (58.8% LSV-A reads in pool 3C and 59.4% acute bee paralysis virus [ABPV] reads in pool 12C) while being present in negligible quantities (<1% reads) in each of the two remaining replicate samples. This indicates the (sporadic) presence of individual bees with very high viral loads compared to those in other bees within the same honey bee colony.

### Virome comparison with the *Varroa*- and DWV-naive honey bees (Australia).

**FIG 4:**
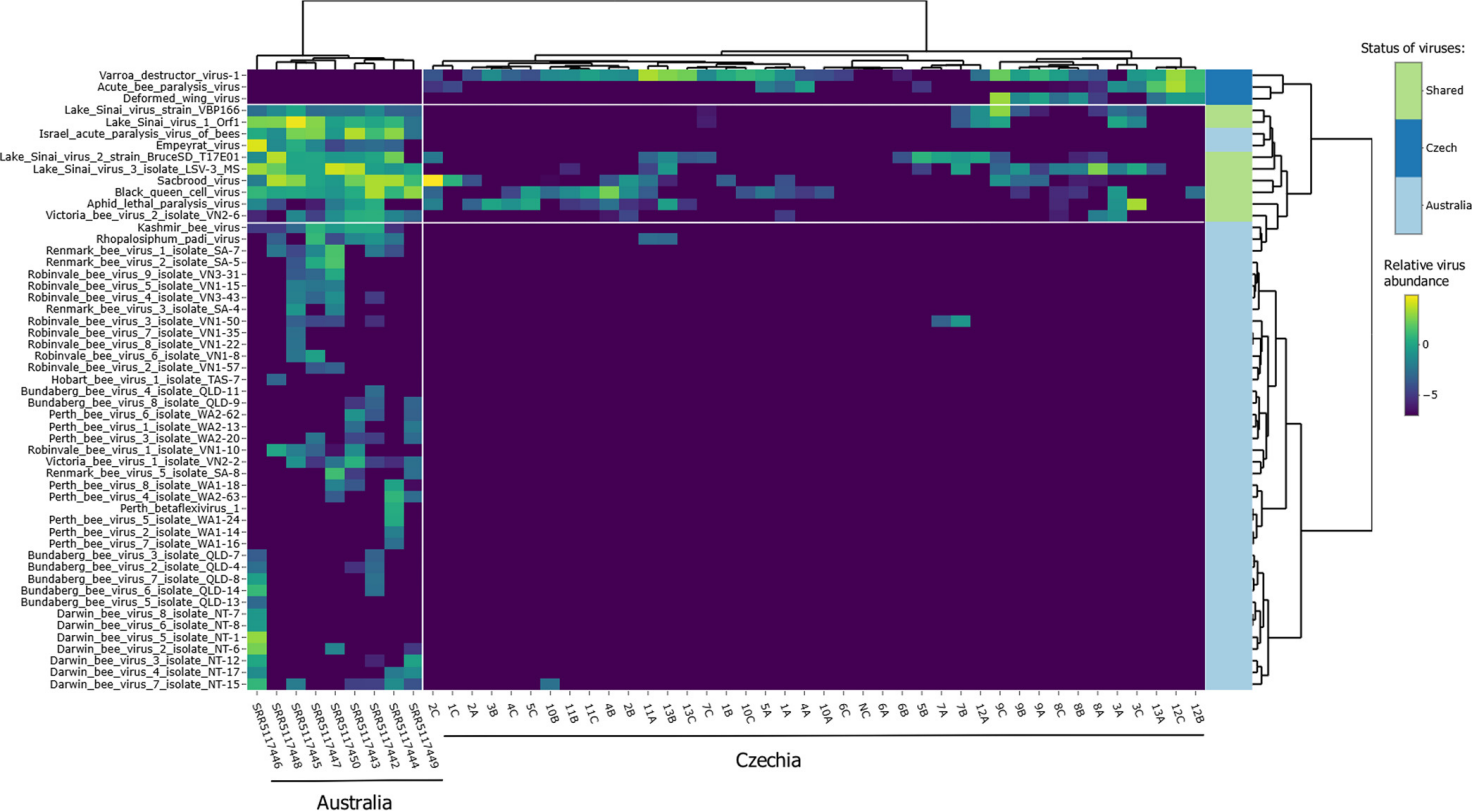
Heatmap of bee-infecting viruses in a comparison between the data from our study and Australian bees (SRA accession numbers SRR5117442 to SRR5117450). Relative abundances are shown on a log_10_ scale. Samples (columns) and viruses (rows) are clustered by Ward’s minimum variance method algorithm and seriated by optimal leaf ordering. Row colors show if the virus is present in Australia, Czechia, or both countries. Czech, viruses found only in Czechia; Australia, viruses found only in Australia; shared, viruses present in both regions. White lines separate the heatmap into several parts, Australian/Czech samples and viruses present/absent in the given regions.

Since we used healthy asymptomatic bees where varroosis was under control, we compared our data with those reported previously by Roberts et al. (16). The heatmap in Fig. 4 shows that the virome of *Varroa-* and DWV-naive Australian honey bees is different from that of the Czech samples. All the Australian viromes clustered together and were separated from the Czech viromes. The Australian viromes included several abundant and diverse viruses belonging to the *Picornavirales* (e.g., Perth bee virus, Darwin bee virus, or Robinvale bee virus). In contrast, we did not detect any of the diverse *Picornavirales* (Fig. 4). Conversely, DWV-A/B and ABPV found in Czech honey bees were absent in the nine Australian viromes. Finally, several viruses (BQCV, variants of LSV, SBV, and ALPV) were present in both Australian and Czech viromes.

Prompted by these results, we decided to investigate if this difference was also discernible in other studies of non-Australian viromes. We used public NGS data from Belgium (34); Israel (30); South Africa, The Netherlands, and Tonga (29); and the United States, Central America, Europe, Kenya, India, and New Zealand (26). This allowed us to compare the bee populations where *V. destructor* is present (most of the world) with *Varroa-*naive populations (16). The results showed that the difference observed between the Czech and Australian bee viromes can be generalized to other parts of the world: the above-mentioned diverse *Picornavirales* were absent from the honey bee viromes in all other geographic regions except Australia (the trace amount can be attributed to read misalignment), whereas DWV-A and DWV-B were globally abundant. Australia clustered aside from other interleaved samples (for the principal-coordinate analysis [PCoA] and heatmap, see Fig. S1; for raw data, see Table S3).

10.1128/msystems.00072-22.6 TABLE S3 Raw data from comparison of Australian and other viromes, including accession numbers used for viral genomes. Download Table S3, XLSX file, 0.05 MB. Copyright © 2022 Kadlečková et al. 2022 Kadlečková et al. https://creativecommons.org/licenses/by/4.0/ This content is distributed under the terms of the Creative Commons Attribution 4.0 International license.

### Emerging viruses in samples from Czechia.

The heatmap of bee virus abundances (Fig. 3) revealed a conspicuous pattern of cooccurrence of two recently discovered rhabdoviruses, bee rhabdovirus 1 (BRV-1) and BRV-2, which possibly infect both the honey bee and the mite *V. destructor* (29). Among our samples, BRV-1 and BRV-2 were always present together (pools 4A, 6A, 7C, 9A, and 10A), whereas both identified rhabdoviruses were absent from the remaining replicates (Fig. 3). The positivity of one out of three replicates probably implicates a low prevalence of BRV-positive bees within hives. In addition, BRV-1 always showed a higher abundance than BRV-2 in individual samples (Fig. 3). These viruses, albeit related, are phylogenetically distinct and display very limited sequence similarity (Fig. S1).

Furthermore, we reanalyzed NGS data from BRV-1-positive samples reported in three previous studies (26, 29, 35) by differentially mapping the sequencing reads to BRV-1 and BRV-2 reference genomes. Despite differences in multiple sample characteristics (i.e., bee pooling, nucleic acid isolation, and library preparation), we detected BRV-2 in all BRV-1-positive samples (Table 1). As in the Czech samples, BRV-2 was always present at a lower abundance. We consider this to be an indicator of an unusual relationship between the two rhabdoviruses, which has been left unnoticed previously (see Discussion).

**TABLE 1:** Cooccurrence of bee rhabdoviruses 1 and 2 in NGS samples here and in three other studies where BRV-1 was identified^*a*^

Sample ID	No. of BRV-1 reads	No. of BRV-2 reads	BRV-1/BRV-2 ratio	Country	SRA accession no.	No. of bees/sample	NA for library construction
SRR3927497	9,509	765	12.4	Israel	SRR3927497	30	Total RNA
DWV	41,335	1,024	40.4	USA	SRR6033679	10	Virus-enriched (encapsulated) DNA + RNA
NE_AWD_1442	34,115	737	46.3	The Netherlands	SRR5109823	5	Total RNA (rRNA depleted)
SA_RI_49	83,757	6,791	12.3	South Africa	SRR5109831	5	Total RNA (rRNA depleted)
T_V9	104,233	181	575.9	Tonga	SRR5109822	5 (thoraces only)	Total RNA (rRNA depleted)
T_V10	38,738	12,120	3.2	Tonga	SRR5109821	5 (thoraces only)	Total RNA (rRNA depleted)
T_T12	449,293	4,501	99.8	Tonga	SRR5109828	5 (thoraces only)	Total RNA (rRNA depleted)
T_T23	331,169	113	2,930.7	Tonga	SRR5109834	5 (thoraces only)	Total RNA (rRNA depleted)
4A	44,492	6,741	6.6	Czechia	SRS11094606	9	Virus-enriched (encapsulated) DNA + RNA
6A	83,517	4,475	18.7	Czechia	SRS11094614	9	Virus-enriched (encapsulated) DNA + RNA
7C	7,029	1,072	6.6	Czechia	SRS11094620	9	Virus-enriched (encapsulated) DNA + RNA
9A	127	40	3.2	Czechia	SRS11094624	9	Virus-enriched (encapsulated) DNA + RNA
10A	16,770	1,493	11.2	Czechia	SRS11094627	9	Virus-enriched (encapsulated) DNA + RNA

a Sequencing reads were mapped to a hybrid reference sequence consisting of combined BRV-1 and BRV-2 genomes (BRV-1, GenBank accession number MH267692; BRV-2, GenBank accession number KY354234) to prevent the interference of multiple mapped reads. The BRV-1/BRV-2 ratio was calculated from read counts.

**FIG 5:**
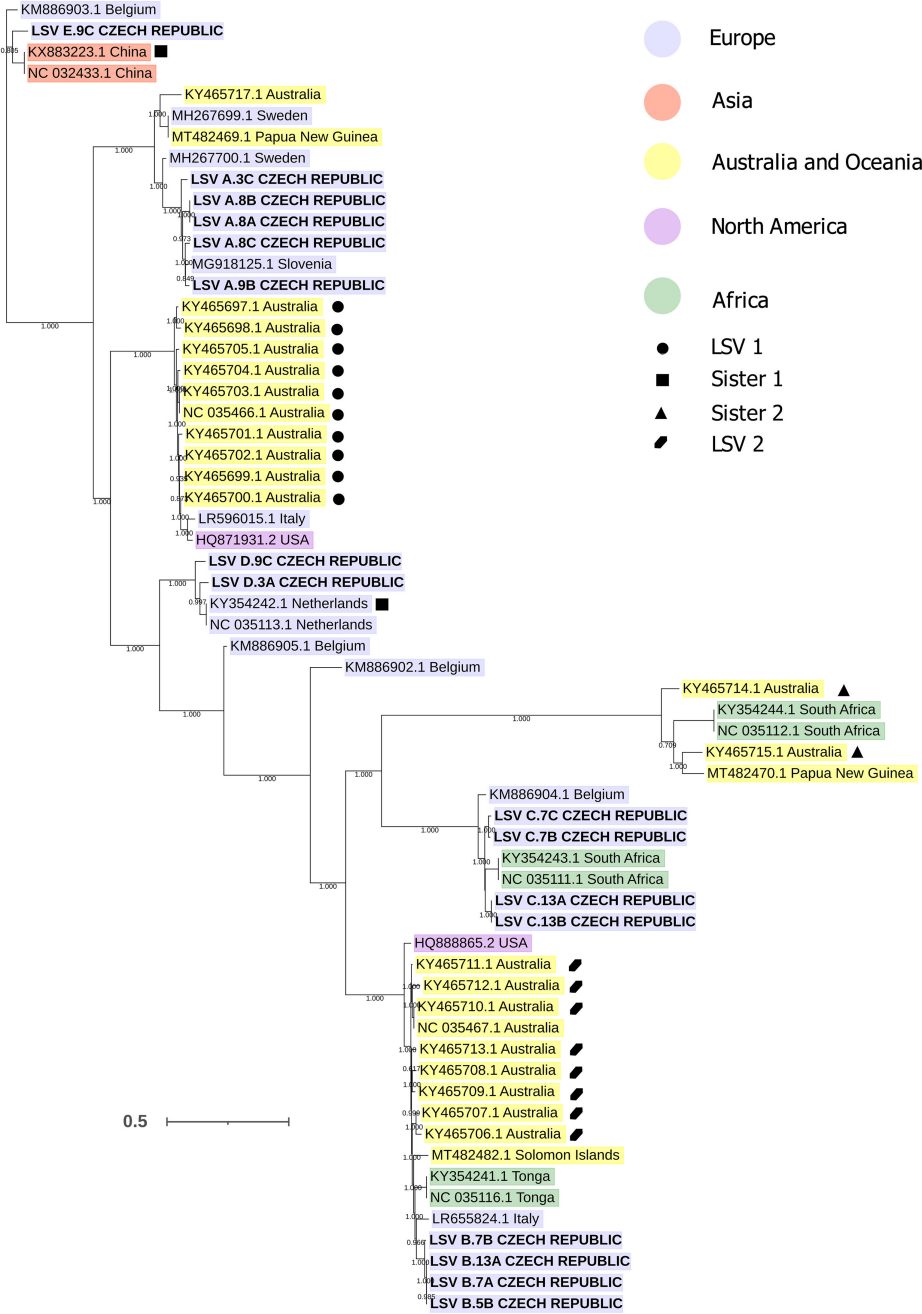
Phylogenetic tree of full LSV genomic sequences. LSV sequences are highlighted in color according to the continent of origin. Symbols mark isolates assigned to LSV lineages by Cornman (72). Sequences obtained in this study are in boldface capital letters. Bootstrap values are shown.

Surprisingly, our analysis also revealed a large diversity of LSVs. The LSV genomes identified in the Czech samples were distributed in five positions in the global LSV phylogenetic tree (Fig. 5). Five variants were thus designated *de novo* as LSV-A to LSV-E for the purpose of this study. The interlineage nucleotide identity among Czech genomic sequences ranged from 72% to 79%, and the intralineage identity ranged from 86% to 99%. As shown in Fig. 5, among the closest relatives of LSV variants A, B, C, and E were sequences originating from different continents, suggesting that these variants belonged to LSV lineages with an intercontinental or even a global distribution. The RNA-dependent RNA polymerase (RdRp) phylogeny places LSV-D among a dozen European and one Iranian LSV genotypes; however, the resolution of the RdRp fragment phylogeny is insufficient, as indicated by the level of bootstrap support (Fig. S2).

10.1128/msystems.00072-22.2 FIG S2 Phylogeny of RdRp regions of LSVs. Download FIG S2, PDF file, 0.3 MB. Copyright © 2022 Kadlečková et al. 2022 Kadlečková et al. https://creativecommons.org/licenses/by/4.0/ This content is distributed under the terms of the Creative Commons Attribution 4.0 International license.

In general, the distribution of LSV variants in Czechia was variable in both between- and within-hive comparisons. Interestingly, two or more variants of LSV were detected in individual hives (LSV-B and LSV-C in hives 7 and 12 and LSV-A, LSV-D, and LSV-E in hive 3).

### Bacteriophages and clustering.

**FIG 6:**
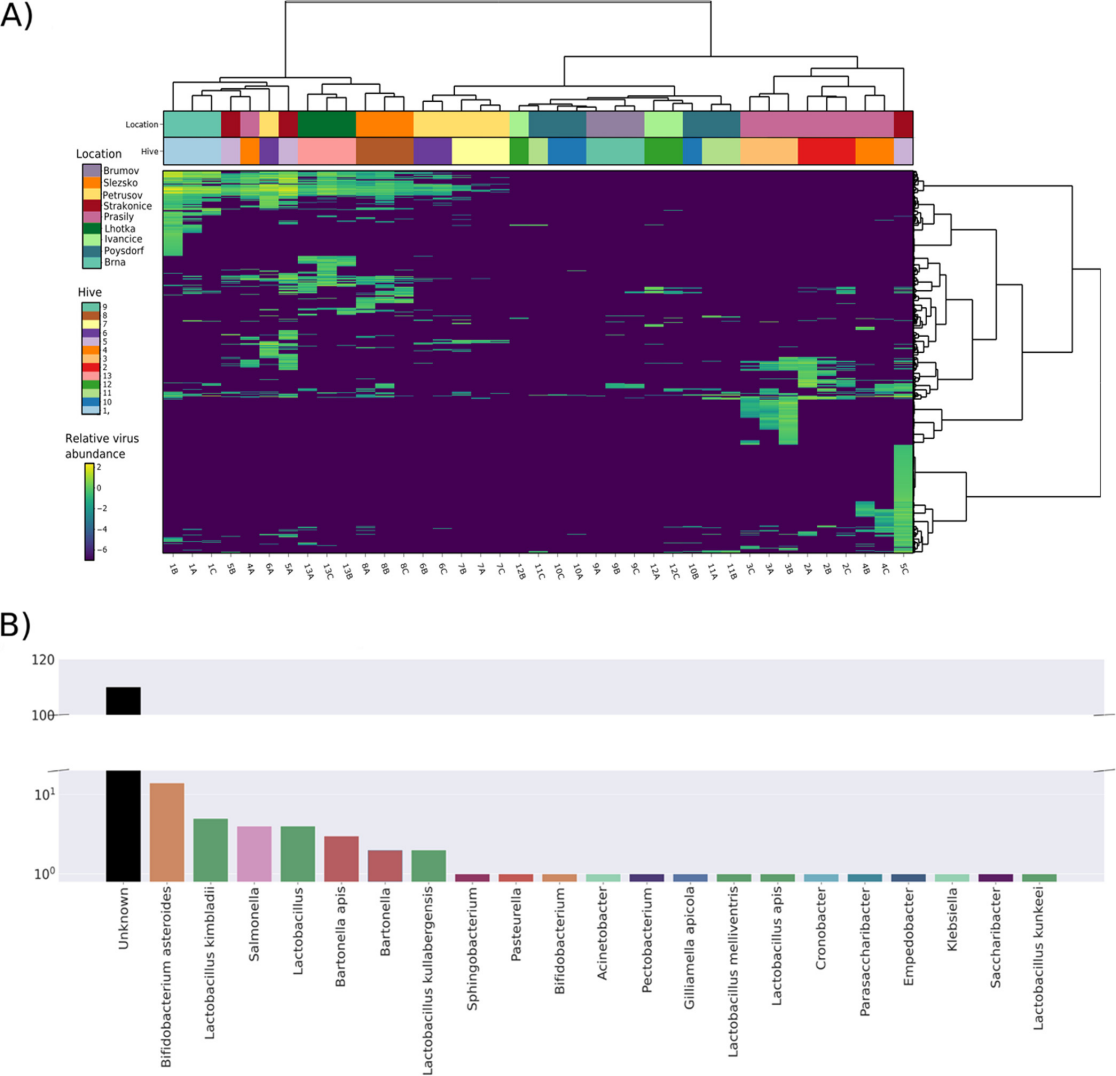
Host calling and clustering of prokaryotic viruses. (A) Heatmap of all contigs classified as bacteriophages. Relative abundances (viruses per 1 million sequencing reads) are shown on a log_10_ scale. Samples (columns) and contigs (rows) are clustered by Ward’s minimum variance method; both columns and rows are seriated by optimal leaf ordering. (B) Count of predicted hosts for 158 bacteriophage contigs identified by VirSorter2. Contigs with no prediction are in the “Unknown” category.

The results showed the high diversity and abundance of bacteriophages (Fig. S1). Therefore, we analyzed the bee phageome in detail. A heatmap was created by mapping reads to the set of phages identified by VirSorter2 (Table S1). Clustering of the phageome by the geographic origins of the samples was weaker than that in the heatmap of all viral sequences (Fig. 2), but still, replicate samples from seven hives clustered together. In five other hives, two out of three replicate samples clustered together; the replicate samples from hive 13 did not cluster (Fig. 6A). The Adonis test showed a highly significant correlation between the phageome and hive/location (*P* < 0.0001 by an Adonis-Bray test; *R*^2^, 0.47355/0.34024).

Since the plant viruses originating from pollen were abundant (Fig. S1), we further explored their clustering. The plant viruses clustered almost perfectly (Fig. S1) (*P* < 0.0001 by an Adonis-Bray test; *R*^2^, 0.70959 for hive and 0.53102 for location). Interestingly, k-means clustering had higher adjusted mutual information scores for both bacteriophages and plant viruses than for all viral sequences (0.22 and 0.23, respectively, against 0.14) (Fig. S1).

To classify the phage genomes, which were predicted to be more than 50% complete (by CheckV), we used vConTACT2, which clustered the sequences with phages in the RefSeq database by their encoded protein profile. The resulting network had 398 individual viral clusters (roughly equivalent to genus-level assignment). Visualization of the resulting sequence similarity network (Fig. S3) shows the distribution of putative phage contigs through the network. Out of 158 individual phage contigs, 71 were unambiguously clustered. These formed 22 clusters, 15 of which were composed entirely of putative phage contigs from this study; 22 viral clusters (representing 26 putative bacteriophage contigs) were clustered with at least one reference sequence. Thanks to the reference sequences, the clusters could be tentatively classified as belonging to the family *Myoviridae* but also as belonging to the *Siphoviridae*, *Podoviridae*, and *Microviridae*. A single cluster contained strains from more than one viral family.

10.1128/msystems.00072-22.3 FIG S3 Similarity network for bacteriophages. Download FIG S3, PDF file, 0.2 MB. Copyright © 2022 Kadlečková et al. 2022 Kadlečková et al. https://creativecommons.org/licenses/by/4.0/ This content is distributed under the terms of the Creative Commons Attribution 4.0 International license.

Host calling for each of the 158 detected phage contigs was performed through matches with CRISPR spacers identified using MinCED (36) and an additional analysis with CrisprOpenDB (37). Over 200 spacers matched the detected viral contigs, yet due to duplicate assignments (one contig matching spacers from multiple strains of one bacterial host species), only 22 (14%) of the phage contigs could be assigned to a host. The most common phage hosts were *Lactobacillus* species, *Bifidobacterium*, *Bartonella*, and Salmonella (Fig. 6B).

## DISCUSSION

This study is the first to analyze the honey bee virome in Czechia, a country located in the heart of Central Europe. In addition, to our knowledge, this is the first work where the honey bee virome was examined in biological replicate samples within hives/colonies. Virome variation in sample replicates raises the question of to what extent single bees can affect the virome structure of an entire colony. However, the open question that remains is how NGS-based virome analysis of pools of bees can be affected by a single bee with a distinct virome. It is relevant to what we have attempted: to examine the homogeneity of virus infection within hives. A major factor that could affect the virome is that honey bee colonies were from a Central European country with one of the highest colony densities worldwide (10). Thus, despite the fact that *V. destructor* occurrence was low in all investigated colonies, and no signs of varroosis were observed, a virus(es) introduced by the mite (17, 38) was expected to be found. Furthermore, we compared our viromes with previously described viromes of Australian honey bees, which have never been exposed to varroosis (DWV and *Varroa* mite) (16, 39). When it came to bee-infecting viruses, our sets of viruses were diametrically different from those in Australia. Notably, one substantial difference was the lack of diverse members of the *Picornavirales* in our data set, which could be explained by *Varroa*-DWV interaction pressure indicated previously (17–22). Further investigation will be necessary because of methodological differences in this study compared to the study of Roberts et al. (16). For future comparability with other studies, it is also of importance to note that our samples were collected at the end of the beekeeping season in August/September because seasonal variations in virus occurrence have been previously described (40), whereas the Australian samples were collected between a longer period (August 2013 to April 2015, but the majority were collected in August).

### Traditional and new bee viruses.

The following viruses were detected in Czech samples: BQCV, AmFV, DWV-A and -B, SBV, ALPV, ABPV, BRV-1 and -2, Apis mellifera orthomyxovirus, and variants of LSV. The prevailing honey bee virus, in both abundance and prevalence, was DWV-B. Importantly, this result is in accordance with its recent global spread (20). Notably, the prevalence of DWV-B observed in asymptomatic (healthy) hives/colonies is in agreement with the results of a study by Norton et al., who showed DWV-B persistence in colonies with low *V. destructor* levels and those treated with miticides (21). Finally, the prevalence of DWV-A was low in our virome samples, consistent with the fact that varroosis was under control in our colonies (21).

We determined the viromes in 39 pools, each consisting of nine individual honey bees. Importantly, we show that none of the structures of our Czech virome resembled those of the Australian viromes (16). Moreover, unique viruses were identified in each data set. These results may correspond to the fact that the virome structure is affected by the presence/absence of *V. destructor* since Australian honey bees are *Varroa*-free (16, 39), while the Czech viromes originated from regions where *V. destructor* has been widely distributed since the late 1970s (41, 42). Although we worked with “healthy” honey bee colonies, where *V. destructor* was under control, the effect of the mite is still to be considered. In Czech samples, the diverse set of viruses belonging to the *Picornavirales* detected in Australian honey bees was absent. Another important observation is the complete absence of DWV in the Australian viromes (16) and the contrary wide DWV presence in our samples. This shift from diverse *Picornavirales* members to a primarily DWV-dominated virome could signify that the presence of *V. destructor* and its interaction with DWV change the virome of honey bees. We suggest that the absence of diverse *Picornavirales* in other samples from honey bee populations around the world, even though varroosis was under control in some, adds support to this assumption.

The two rhabdoviruses (BRV-1 and -2) exhibited a conspicuous pattern of distribution among our samples: (i) both viruses were always present simultaneously; (ii) in each sample, BRV-1 was more abundant than BRV-2; and (iii) BRV-1 and -2 were always present in only one out of three replicate nine-bee samples per hive. Reanalysis of previously reported BRV-positive NGS samples (26, 29, 35) independently validated both the first and second phenomena (see above). So far unnoticed, this relationship may indicate a type of (inter)dependence between these two viruses. It is possible that an interaction takes place, either a nondirect one (e.g., through the immune system) or a direct one (e.g., a defect in protein synthesis) (43). This phenomenon can also be caused by a common transmission route or another unknown mechanism. Either way, the precise nature of the relationship between BRV-1 and BRV-2 should be confirmed by a single-bee analysis.

Since the first description of LSVs by Runckel et al. (44), various variants of this group of new viruses have been discovered in different regions around the world, including Australia (39, 45–48). Such diversity, in combination with a global distribution, could indicate a long coexistence of LSV with honey bees (39). According to current knowledge, LSV infections occur asymptomatically, with no described adverse effect, which might have hindered their discovery in the pre-omics era. It was suggested that, based on the specific antibody detection LSV was described as bee virus X or Y (39, 49, 50). Among 39 Czech samples, we detected five distinct variants (designated LSV-A to -E) that were distributed in 17/39 samples (read count of ≥1 per million reads). Several cases of the simultaneous presence of several variants in one colony/hive were observed. A single-bee analysis is needed to determine the precise prevalence of LSV variants and the frequency of their cooccurrence as the simultaneous presence of several LSV variants in a single bee has been reported previously (47). The phylogenetic analysis of LSV sequences suggests that the LSV variants identified in the Czech samples belong to lineages that are globally distributed. Given the variable distribution and extraordinary global diversity of LSV, additional LSV variants are likely to be found in future studies. However, the available LSV sequences, both partial and complete, suffer from a heavy geographic sampling bias, implying that the true diversity might be even (much) larger than currently observed.

### Virome structure stability.

When all viral sequences present in our samples were analyzed, most samples clustered in accordance with their site of origin. Clustering was also observed for samples originating from different hives from the same apiary and for hives from different apiaries located in the same municipality. It appears that this clustering is driven mainly by plant viruses and to a lesser extent by bacteriophages (see Fig. S1 in the supplemental material). In this study, bacteriophage sequences were one of the most diverse and abundant virome components in our samples. However, bacteriophages still represent a novel topic in honey bee research (27, 28) and are often excluded from analyses. In the bacteriophage heatmap, replicate samples still clustered based on hive and location, like the whole virome (plant viruses plus bacteriophages and other viral sequences). This is in accordance with previously reported findings that the phageome should be relatively stable in the location for the given year (28). The plant viruses in the total bee virome display the strongest geographic dependence (Fig. S1). Unfortunately, to our knowledge, no studies examining the stability and geographic differences of the pollen virome have been carried out yet.

The robust overall clustering pattern was lost when only viruses known to infect honey bees were analyzed. The uneven distribution of bee-infecting viruses among replicate nine-bee samples from the same hive raises serious questions for the NGS study design. NGS studies are typically carried out on pools of a small number (~1 to 5 [e.g., see reference 34]) or large numbers (>30 [e.g., see reference 35]) of individual bees.

**FIG 7:**
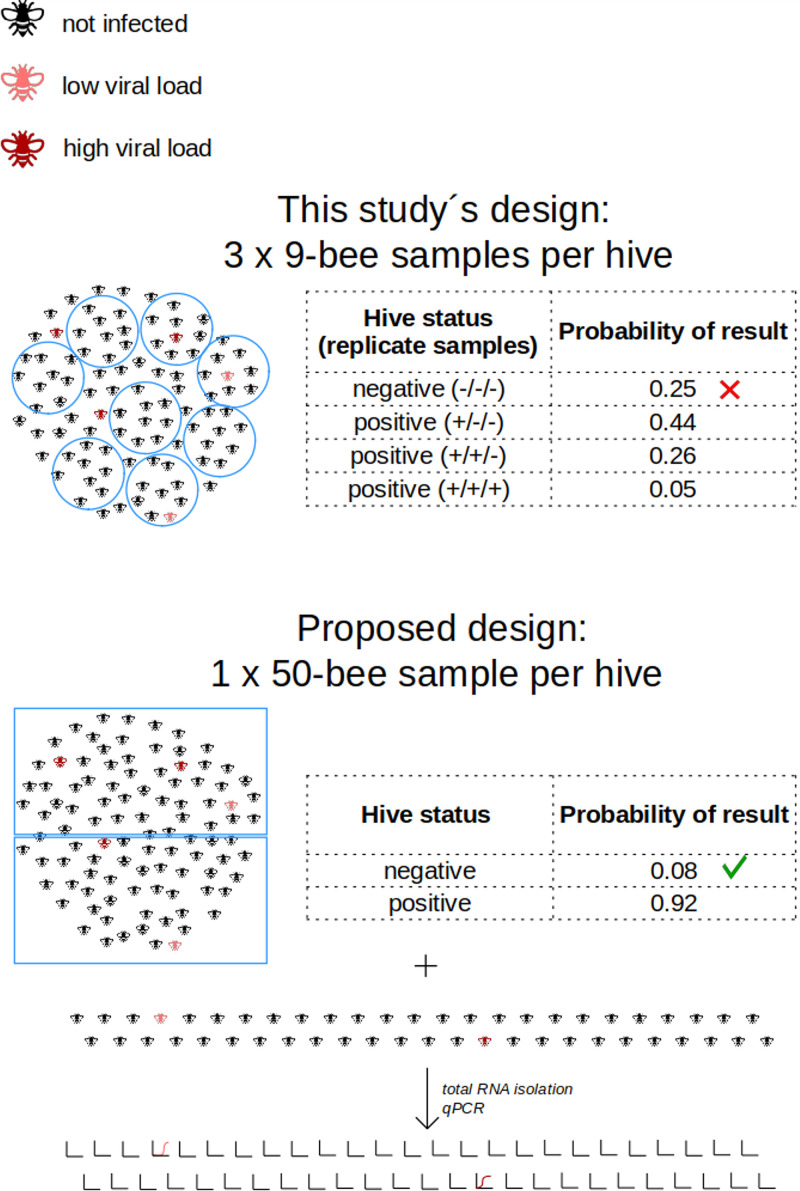
Effect of the NGS experimental design on hive status determination (positive/negative) for low-prevalence bee viruses (5%; 1/20 bees infected). + or − shows positivity or negativity, respectively, for that replicate sample (9-bee sample).

In our case, the pooling of nine bees per sample was not enough to balance the uneven representation of bee viruses among individuals. The recurring observations that only one nine-bee pool out of three yielded positivity for some viruses (e.g., BRV-1/2 [see “Traditional and new bee viruses,” above) indicate that the actual prevalence of infected worker bees per hive is low. In addition, we suspect that individual bees with relatively high viral loads are present in hives, and when they are randomly included in a pool for NGS virome analysis, this virus may dominate the virome of this pool. To accurately determine the abundance and prevalence of bee viruses in a hive, NGS of a large number of libraries, each prepared from an individual bee, appears to be the methodologically correct solution, which unfortunately is labor-intensive, expensive, and, thus, often beyond feasibility. Another possibility is to pool a large number of bees into a single master NGS library per hive (Fig. 7) and carry out NGS at a sequencing depth that is the same as or higher than the one in this study. The actual number of bees per master NGS library pool, our results suggest, should optimally be 50 individuals or more. Such an approach is expected to (i) detect the diversity of the viruses present, including those of low prevalence, and (ii) determine the actual genotypes of the viruses present in an affordable fashion (even though it can be challenging to disentangle closely related genomes). In this case, however, the viral prevalence in hives would remain to be assessed by analyzing similar quantities of individual bees, preferably by a rapid method like quantitative PCR (qPCR) (Fig. 7).

### Bacteriophages.

The phages identified in the Czech viromes belonged to several families, with *Myoviridae*, *Podoviridae*, *Microviridae*, and *Siphoviridae* being the most common. Out of the total of 158 (at least 50% complete) phage genomes, 26 could be directly clustered with the reference sequences from the database (approximately genus-level assignment), while the majority of contigs could not be assigned to a known genus or family (132), suggesting that multiple novel phage genera are present in honey bees. Host calling through CRISPR spacers predicted bacterial hosts for around 15% of bacteriophages. The predicted hosts (mainly *Lactobacillus* species, *Bifidobacterium*, *Bartonella*, and Salmonella) are those residing in the honey bee gut (51, 52). Even though this assignment level is higher than, e.g., that in the human gut, it did not reach the levels described previously for honey bees (27), with the majority of contigs being assigned to their hosts. This difference may be due to our approach for assigning a host to individual phage sequences instead of a viral cluster or to our stringent setting when comparing CRISPR spacers with phage sequences.

### Conclusion.

In conclusion, we identified an important aspect of the total bee virome: the bee-infecting viruses vary widely among individual bees, while the complete virome, which is composed predominantly of bacteriophages and plant viruses, is largely stable and geographically dependent. As the samples originate from the heart of Central Europe, our virome could be considered representative for the region. We revealed the absence of diverse *Picornavirales* in Czech and other global non-Australian honey bees, probably resulting from the cooccurrence/interaction of *V. destructor* and DWV. We report the wide presence of LSVs among Czech bees and their unexpected diversity, consisting of five globally represented variants. We provide the first description of a tentative close relationship between two related honey bee-infecting rhabdoviruses. Finally, we provide a preliminary characterization of bacteriophages present in the Czech honey bee virome samples.

## MATERIALS AND METHODS

### Sample origin.

The worker bees were collected from 8 August to 29 September 2018 with owner permission from 13 representative bee colonies of nine beekeepers, some of whom provided more subspecies/subtype information (see Fig. S1 and Table S1 in the supplemental material). The honey bee workers were shaken off the brood combs into plastic bags, which were immediately placed into a box with dry ice for transport. The samples were then stored at −80°C until analyses. An overview of sampling sites (13 hives, 11 apiaries, and 9 locations) is shown in Fig. S1. All apiaries enrolled in this study were healthy; i.e., they did not exhibit symptoms of common pathogen infections or *V. destructor* infestation. All hives (except hive 9) were previously treated against *V. destructor* by either organic acids or amitraz. Three subspecies/subtypes of honey bee as provided by the beekeeper were included: Buckfast honey bee (*A. m. buckfast*), a hybrid of dark honey bee (*A. m. mellifera* Linnaeus 1758), and Carniolan honey bee (*A. m. carnica* Pollman 1879). Some bees originated from different apiaries from the same location or from different hives of the same apiary (metadata are available in Table S1).

### Nucleic acid extraction and sequencing library preparation.

Bees were processed according to the NetoVir protocol (28, 53). Three randomly chosen bees from each hive were homogenized in a tube with 2.8-mm ceramic beads (zirconium oxide) (Bertin Technologies, Montigny-le-Bretonneux, France) and 1 mL of sterile phosphate-buffered saline (PBS) using a Minilys homogenizer (Bertin Technologies, Montigny-le-Bretonneux, France) at 3,000 rpm for 5 min. After centrifugation at 17,000 × *g* for 3 min, the supernatant was filtered through a 0.8-μm filter (polyethersulfone [PES]) (Sartorius, Göttingen, Germany) and centrifuged again at 17,000 × *g* for 1 min. The supernatant from three homogenates, each consisting of three individuals, gave a pool of nine bees after mixing in one tube. Overall, three distinct replicates of the pooled nine individuals from a hive, denoted replicates A, B, and C, were used in further analyses.

For nuclease treatment, 260 μL of each pooled sample was used and treated with 4 μL of Benzonase nuclease and 2 μL of micrococcal nuclease (New England BioLabs, Ipswich, MA, USA). The total nucleic acids were extracted using the QIAamp viral RNA minikit (Qiagen, Hilden, Germany) according to the manufacturer’s instructions, without using carrier RNA. Extracted nucleic acids were reverse transcribed and amplified with the WTA2 kit using 17 amplification cycles (Sigma-Aldrich, St. Louis, MO, USA). The concentration of samples was measured by the Qubit dsDNA (double-stranded DNA) HS (high-sensitivity) assay kit (Thermo Fisher Scientific, Waltham, MA, USA) on the Qubit 2.0 fluorometer. Three nanograms of isolated DNA was processed with the Nextera XT library preparation kit (Illumina, San Diego, CA, USA). The quality of the DNA libraries and size distribution were evaluated using a high-sensitivity DNA assay on a Bioanalyzer 2100 instrument (Agilent Technologies, CA, USA), and the concentration was measured on the Qubit 2.0 fluorometer. The libraries were sent on dry ice to the KU Leuven Nucleomics Core (VIB), Leuven, Belgium, for analysis. Sequencing was performed on the HiSeq2000 platform (Illumina, CA, USA) for 2× 150-bp paired-end cycles.

### Bioinformatic analysis. (i) Sequencing data processing and assembly.

Sequencing quality was assessed using FastQC (Babraham Bioinformatics, Cambridge, UK) both before and after trimming. Adapters and low-quality bases were removed using Trimmomatic (54) with settings of 4, 20; leading of 19; tailing of 15; and minlen of 50. The assembly of trimmed reads was done with SPAdes (55) with the metagenomic option and using the following k-mers: 21, 33, 55, and 77. Contigs larger than 500 nucleotides (nt) and with identities of >95% and coverage of at least 80% of the length of the shortest scaffold were merged with ClusterGenomes (https://bitbucket.org/MAVERICLab/docker-clustergenomes). DIAMOND (56) with the settings -sensitive and -c 1 was used to compare sequences against the nonredundant protein database (NCBI) downloaded on 30 September 2018 and subsequently annotated via Kronatools (57). Individual reads were mapped against the nonredundant contigs with BWA-MEM (58), and BamM was used to determine coverage (tpmean) (https://github.com/Ecogenomics/BamM).

### (ii) Targeted analysis of bee-infecting viruses.

To obtain precise mapping information about the viruses that infect honey bees, we performed an additional analysis targeted on individual bee viruses. Reference genomic sequences of all currently recognized viral species known to infect honey bees (33) were retrieved from the GenBank database. Sequencing reads were mapped to these reference sequences under conditions of a maximum of 20% mismatches and a maximum of 20% gaps using the Geneious 6.0.3 Read Mapper (59). Consensus nucleotide sequences (majority rule) were called for viruses with complete or nearly complete coverage of reference sequences; the terminal and low-coverage regions of consensus sequences were visually inspected and manually curated. Virus abundance values (virus reads per 1 million sequencing reads) were determined from the sequencing read coverage of the actual viral sequences present in the samples from Czechia.

### Comparison with other studies.

For comparisons, we addressed data from previous honey bee virome studies (16, 26, 29, 30, 34). FastQ files were retrieved with the prefetch and fasterq-dump tools available in the SRA toolkit (NCBI). Reads were mapped against viruses known to infect bees (33) with BWA-MEM (58), and coverage was extracted with BamM using the tpmean method. Only RNA viruses (DNA viruses were not investigated in the Australian samples) with sum tpmean values over all samples of ≥20 were included for downstream analysis. Furthermore, we specifically screened our data for novel viruses from the order *Picornavirales*.

### Phylogenetic analysis.

For phylogenetic analysis, complete and partial (>500-bp; RdRp) LSV sequences (as of 6 January 2021) were retrieved from the NCBI database and combined with the LSV sequences obtained in this study. Sequence alignment was done with MAFFT with –localpair; –maxiterate 1000 (60). Alignments were trimmed with trimAL -automated1 (61), and the best model was determined by ModelTest-NG (62). Phylogenetic trees were created with PhyML (63) with the model best suited for alignment.

### Bacteriophage identification.

Bacteriophages were identified from all nonredundant contigs (>500 bp) with VirSorter2 (64), ignoring the groups Nucleocytoplasmic large DNA viruses and *Lavidaviridae*. Presumed phage contigs were checked with CheckV (65) to determine their “completeness.” Putative phage sequences that were at least 50% complete were then classified with VConTACT2 (66) with the BLASTP mode using the Prokaryotic Viral RefSeq 88 database MCL for protein clustering and ClusterONE for genome clustering. Host calling was performed using CRISPR spacers retrieved from a set of 304 genomes of bacterial species described to reside in the honey bee gut (NCBI and JGI IMG/M) (Table S2). Bacterial genomic sequences were processed with MinCED (36), and the predicted spacers were pulled and analyzed by BLAST against phage contigs with the stringent settings -ungapped and -perc_identity 100. A complementary host-calling approach was performed by utilizing a CrisprOpenDB (37) search against all complete bacterial genomes to identify bacterial hosts outside the common spectrum (only level 1 predictions). All predicted hosts from CrisprOpenDB were bee-infecting bacteria less frequently mentioned in the literature and therefore were not included in our database (MinCED). The results of the two predictions were merged.

10.1128/msystems.00072-22.5 TABLE S2 Two sheets showing bacteria that were used for host prediction, one for NCBI and the second for IMG, containing accession numbers and IMG genome identifiers. Download Table S2, XLSX file, 0.01 MB. Copyright © 2022 Kadlečková et al. 2022 Kadlečková et al. https://creativecommons.org/licenses/by/4.0/ This content is distributed under the terms of the Creative Commons Attribution 4.0 International license.

### Statistical analysis and visualization.

Statistical analysis was done in R with the Adonis test (permutations, 10,000) implemented in the vegan package (67). Heatmaps were created using the heatmaply package (68), and trees were visualized with iTOL (69). The VConTACT2 network was visualized in Python with the graph-tool library (70). k-means clustering was done in Python with sklearn (71). For each data set, data were first scaled by a standard scaler, and clusters were predicted with k-means (13 clusters). Labels of these predicted clusters were compared with labels of real clusters (1 to 13). Gained scores are available in Fig. S1.

### Data availability.

The sequencing reads were deposited in the NCBI Sequence Read Archive (SRA) under BioProject accession number PRJNA781422. The assembled viral genomic sequences were deposited in GenBank (accession numbers OL803813 to OL803870).
