# Supplementary material for: The Virome of Healthy Honey Bee Colonies: Ubiquitous Occurrence of Known and New Viruses in Bee Populations
Source: mSystems. 2022 May 9;7(3):e00072-22. doi: 10.1128/msystems.00072-22 (PMC9239248; doi:10.1128/msystems.00072-22)
Supplement: FIG S1 [file msystems.00072-22-s0001.pdf]

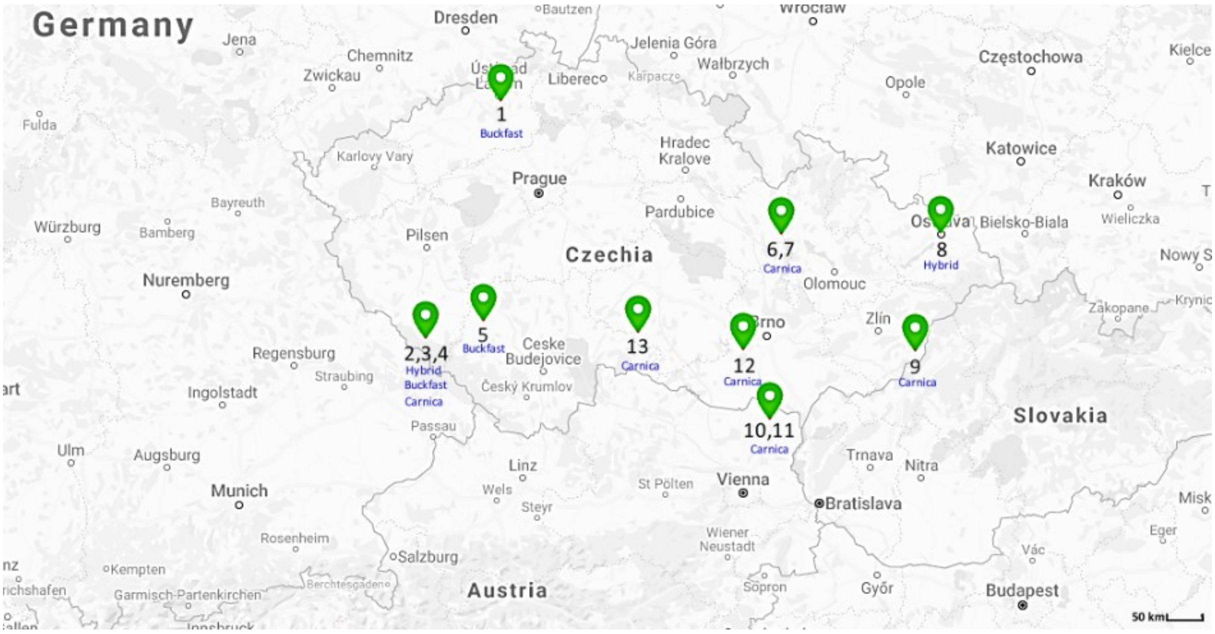

Figure S1.1. Locations of sampling sites with subtypes of honey bees indicated. Samples 2, 3, 4 originated from different apiaries of the same locations while 6, 7 and 10, 11 originated from different hives of the same apiary.

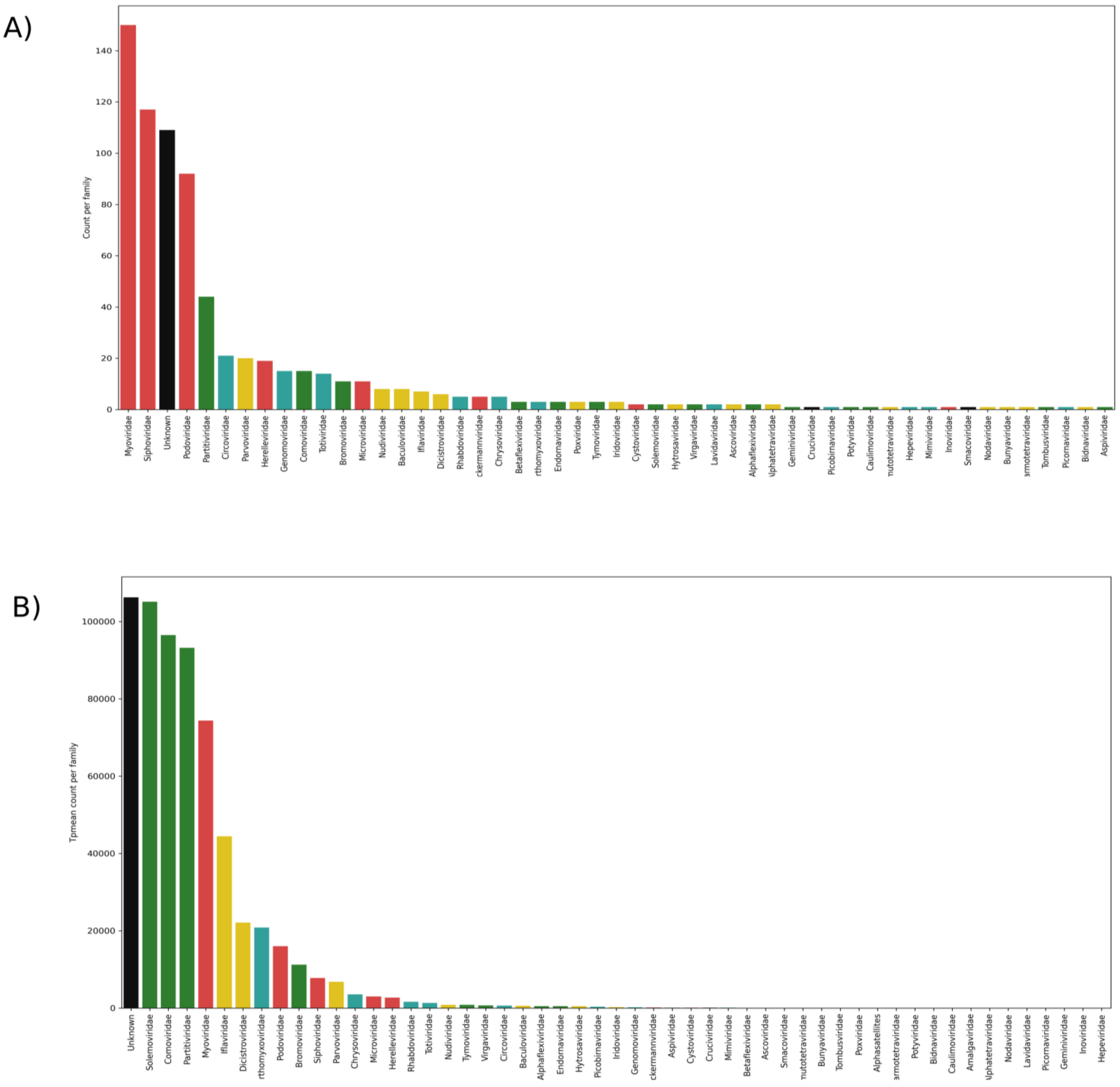

Figure S1.2- Representation of individual viral families in the total honey bee virome. A: number of representatives in individual families after merging on species level, B: Abundances (tpmean) of individual viral families. Viral contigs unassigned at family level are combined in "Unknown" category. Individual columns are coloured based on host specificity of given family (according to ViralZone). Black: Unknown, Green: Plant viruses, Red: Bacterial host, Blue: Other/multiple, Yellow: Insect host.

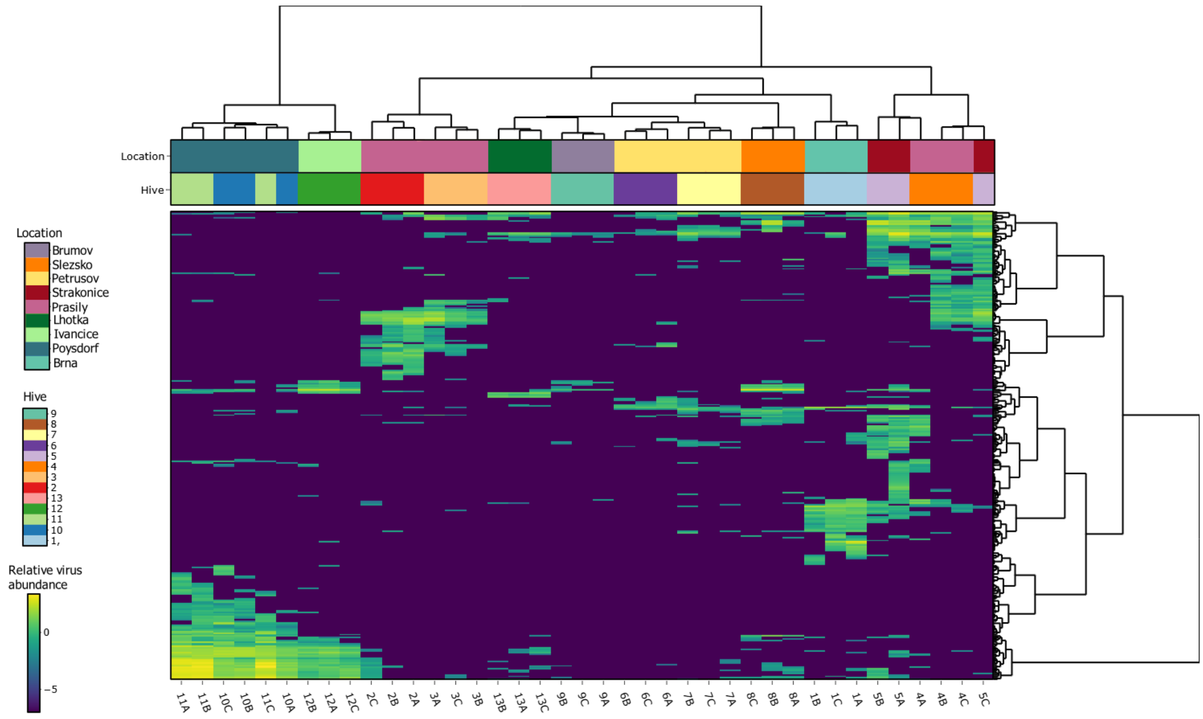

Figure S1.3- Heatmap constructed from all plant viral sequences (families infecting plant host). N = 315. Relative abundances (viruses per 1M sequencing reads) are shown on log10 scale. Samples (columns) and contigs (rows) are clustered by Ward's minimum variance method; both columns and rows are seriated by optimal leaf ordering.

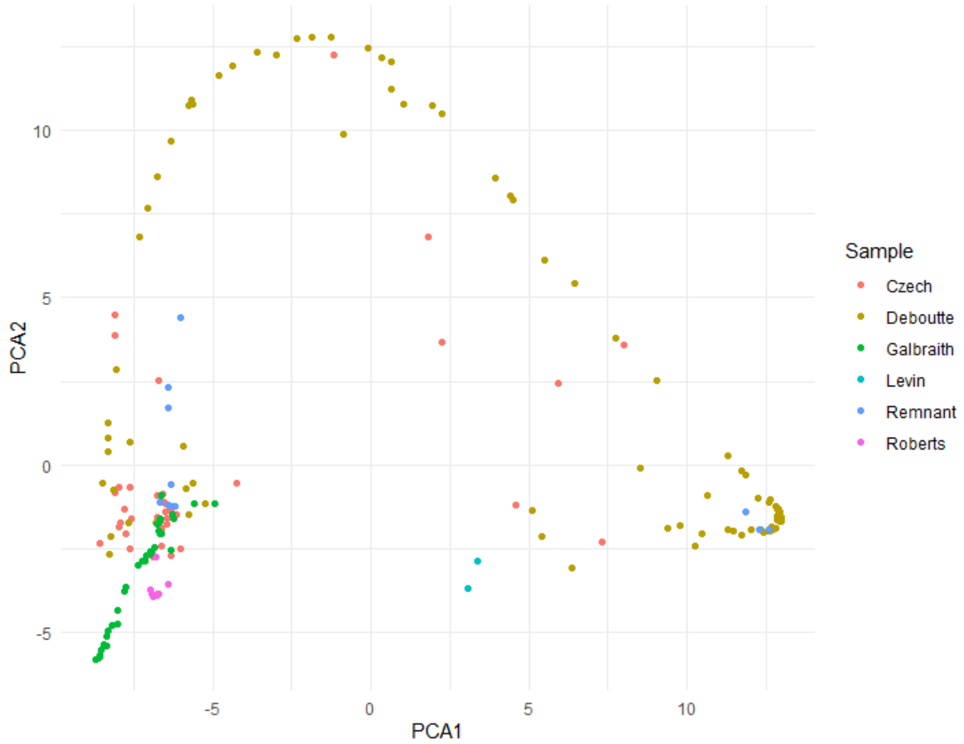

Figure S1.4- PCoA of our samples (Czech) and samples collected around the world (Deboutte, Galbraith, Levin, Remnant) in comparison with Australia (Roberts).

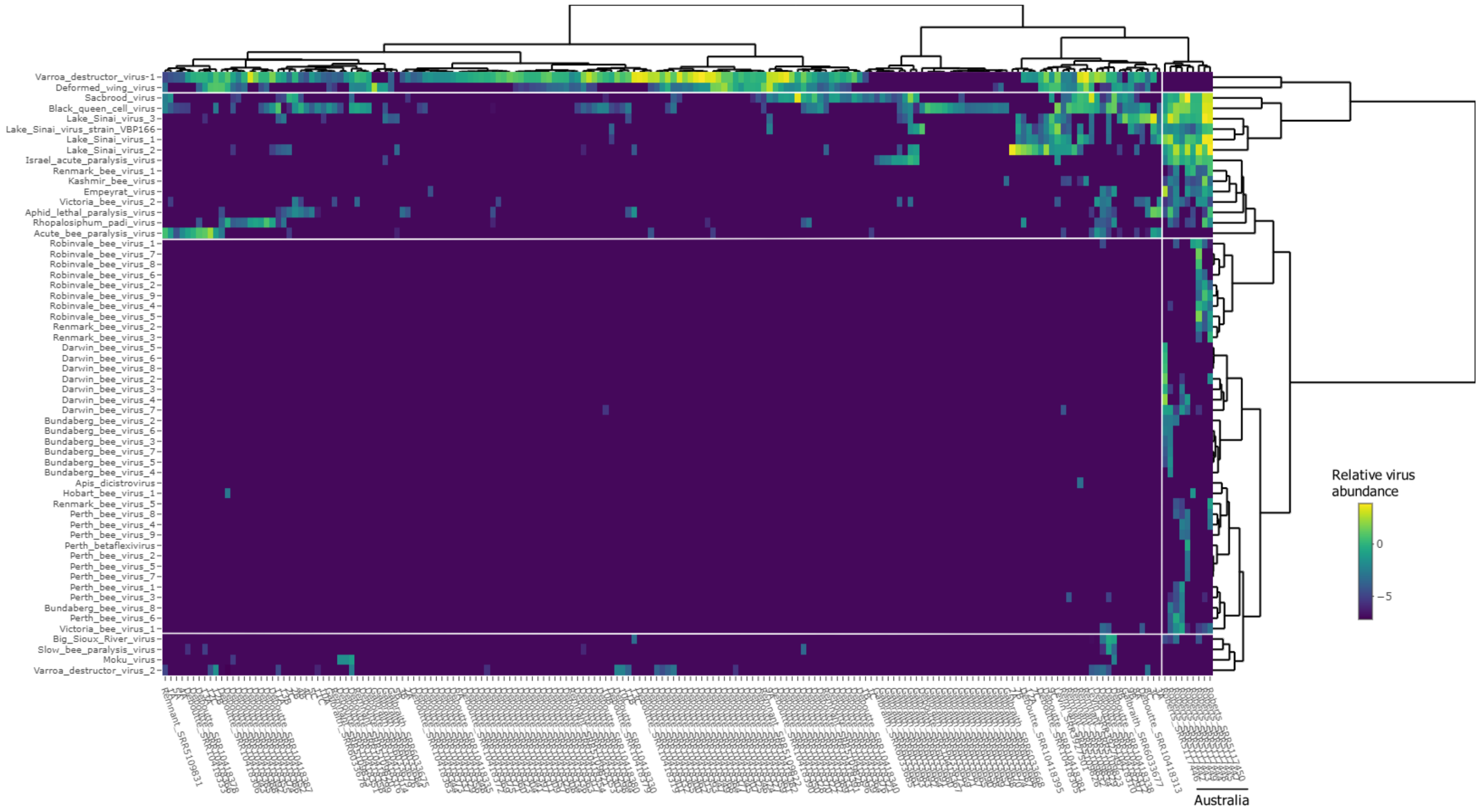

Figure S1.5 – Heatmap of our samples (1A-13C), Australian samples (Roberts), and other studies of Apis mellifera virome (Deboutte, Galbraith, Levin, and Remnant), showing abundances of common and uncommon bee infecting viruses. Relative abundances are shown on a log10 scale. Samples (columns) and viruses (rows) are clustered by Ward's minimum variance method algorithm and seriated by optimal leaf ordering. White lines separate heatmap into several parts. Present/absent/only in Australia and low/high abundant virus.

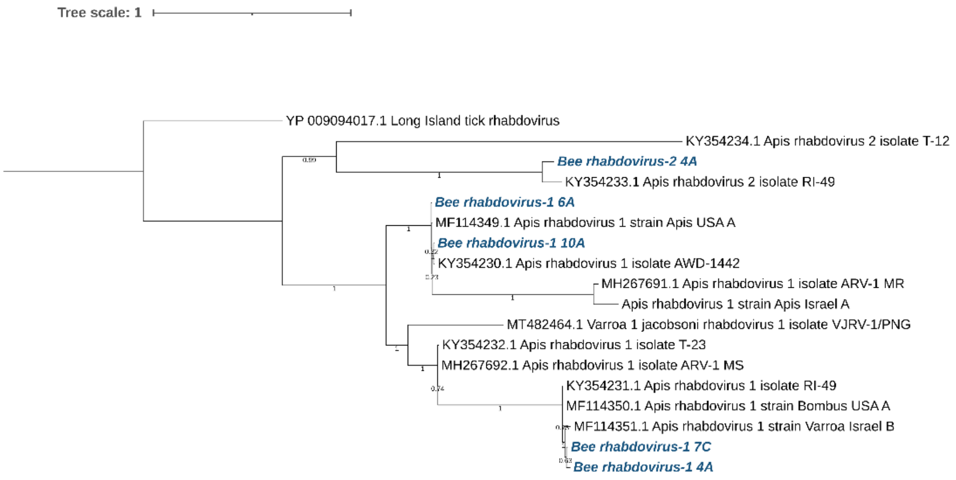

Figure S1.6- Phylogenetic tree of BRV-1 and 2 (aa, L-protein, genes called with Prodigal). Aligned with mafft (--maxiterate 1000 --localpair), trimmed with trimAL (-automated1), best model was determined with Prottest3 and tree build with Phyml. Sequences gained in this study are highlighted in blue.

Figure S1.7- Score of K-means clustering (k 13) for individual groups in our data.

Scores	All viral seq.	Plant viruses	Phages	Bee viruses
Homogeneity score	0.39	0.48	0.45	0.34

Completeness score	0.69	0.73	0.72	0.55
V_measure score	0.50	0.58	0.55	0.42
Adjusted_mutual_info score	0.14	0.23	0.22	-0.01
