# Supplementary figures and images for: The Virome of Healthy Honey Bee Colonies: Ubiquitous Occurrence of Known and New Viruses in Bee Populations
Source: mSystems. 2022 May 9;7(3):e00072-22. doi: 10.1128/msystems.00072-22 (PMC9239248; doi:10.1128/msystems.00072-22)

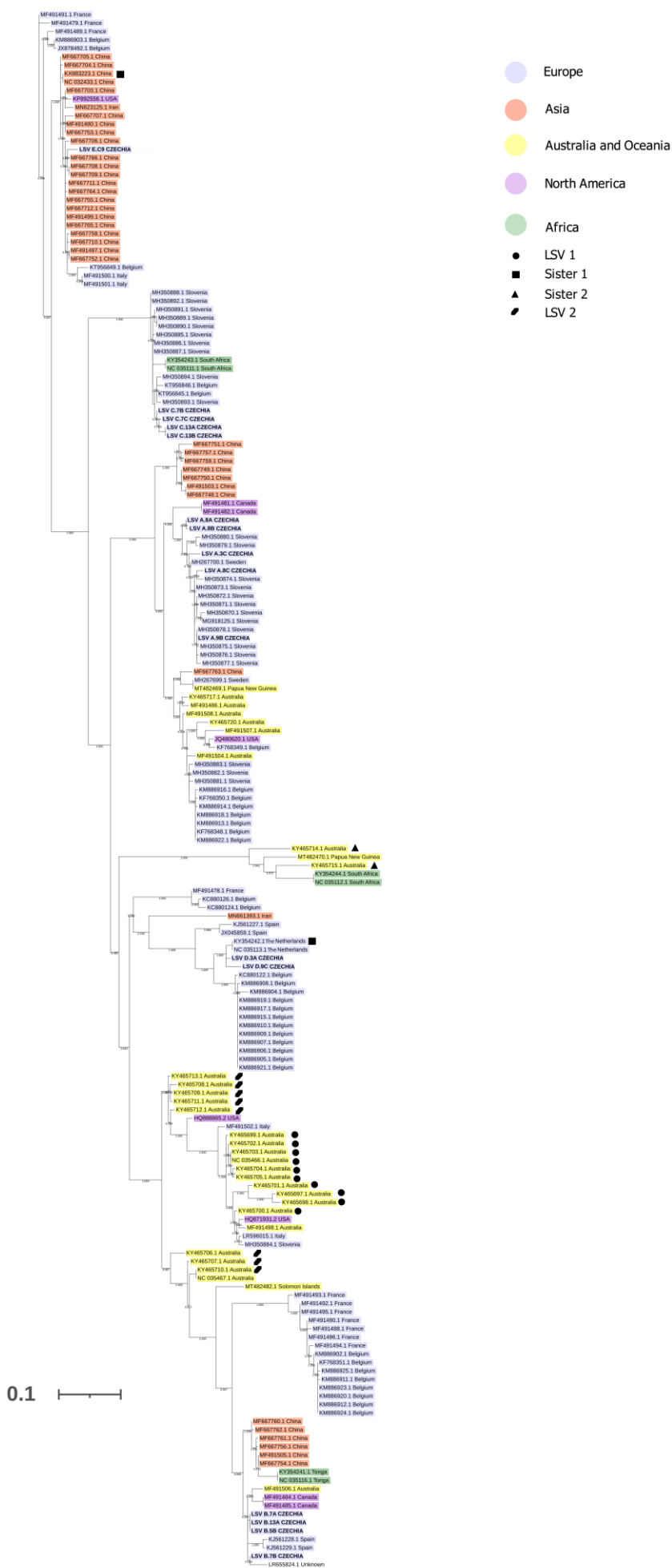

Supplement: FIG S2 [file msystems.00072-22-s0002.pdf]
