# Supplementary material for: The Virome of Healthy Honey Bee Colonies: Ubiquitous Occurrence of Known and New Viruses in Bee Populations
Source: mSystems. 2022 May 9;7(3):e00072-22. doi: 10.1128/msystems.00072-22 (PMC9239248; doi:10.1128/msystems.00072-22)
Supplement: FIG S3 [file msystems.00072-22-s0003.pdf]

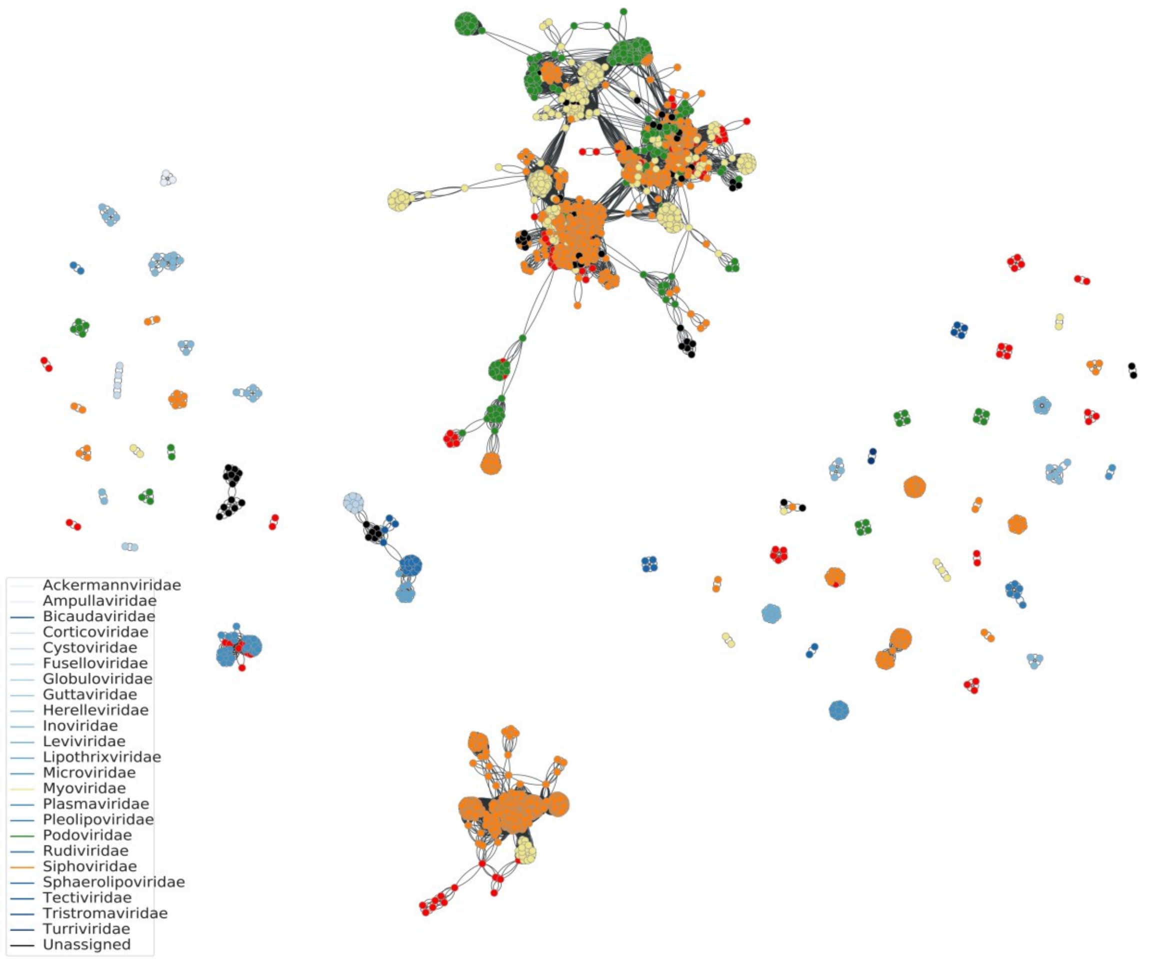

Figure SZ- Similarity network of 158 putative bacteriophage contigs with REFSEQ database. Red colour represents phage contigs while other colours represent family assignment of references.
